# Occurrence of Transferable Integrons and *sul* and *dfr* Genes Among Sulfonamide-and/or Trimethoprim-Resistant Bacteria Isolated From Chilean Salmonid Farms

**DOI:** 10.3389/fmicb.2019.00748

**Published:** 2019-04-12

**Authors:** Mariana Domínguez, Claudio D. Miranda, Oliver Fuentes, Mery de la Fuente, Félix A. Godoy, Helia Bello-Toledo, Gerardo González-Rocha

**Affiliations:** ^1^Laboratorio de Investigación en Agentes Antibacterianos, Departamento de Microbiología, Universidad de Concepción, Concepción, Chile; ^2^Laboratorio de Patobiología Acuática, Departamento de Acuicultura, Universidad Católica del Norte, Coquimbo, Chile; ^3^Centro AquaPacífico, Coquimbo, Chile; ^4^Facultad de Medicina Veterinaria, Universidad San Sebastián, Concepción, Chile; ^5^Departamento de Ciencias Biológicas, Facultad de Ciencias de la Vida, Universidad Andres Bello, Talcahuano, Chile; ^6^Centro i∼mar, Universidad de Los Lagos, Puerto Montt, Chile

**Keywords:** bacteria, sulfonamide resistance, *sul*, integron, salmon farming, Chile

## Abstract

Salmon farming industry in Chile currently uses a significant quantity of antimicrobials to control bacterial pathologies. The main aims of this study were to investigate the presence of transferable sulfonamide- and trimethoprim-resistance genes, *sul* and *dfr*, and their association with integrons among bacteria associated to Chilean salmon farming. For this purpose, 91 Gram-negative strains resistant to sulfisoxazole and/or trimethoprim recovered from various sources of seven Chilean salmonid farms and mainly identified as belonging to the *Pseudomonas* genus (81.0%) were studied. Patterns of antimicrobial resistance of strains showed a high incidence of resistance to florfenicol (98.9%), erythromycin (95.6%), furazolidone (90.1%) and amoxicillin (98.0%), whereas strains exhibited minimum inhibitory concentrations (MIC_90_) values of sulfisoxazole and trimethoprim of >4,096 and >2,048 μg mL^−1^, respectively. Strains were studied for their carriage of these genes by polymerase chain reaction, using specific primers, and 28 strains (30.8%) were found to carry at least one type of *sul* gene, mainly associated to a class 1 integron (17 strains), and identified by 16S rRNA gene sequencing as mainly belonging to the *Pseudomonas* genus (21 strains). Of these, 22 strains carried the *sul1* gene, 3 strains carried the *sul2* gene, and 3 strains carried both the *sul1* and *sul2* genes. Among these, 19 strains also carried the class 1 integron-integrase gene *intI1*, whereas the *dfrA1*, *dfrA12* and *dfrA14* genes were detected, mostly not inserted in the class 1 integron. Otherwise, the *sul3* and *intI2* genes were not found. In addition, the capability to transfer by conjugation these resistance determinants was evaluated in 22 selected strains, and *sul* and *dfr* genes were successfully transferred by 10 assayed strains, mainly mediated by a 10 kb plasmid, with a frequency of transfer of 1.4 × 10^−5^ to 8.4 × 10^−3^ transconjugant per recipient cell, and exhibiting a co-transference of resistance to florfenicol and oxytetracycline, currently the most used in Chilean salmon industry, suggesting an antibacterial co-selection phenomenon. This is the first report of the characterization and transferability of integrons as well as *sul* and *dfr* genes among bacteria associated to Chilean salmon farms, evidencing a relevant role of this environment as a reservoir of these genes.

## Introduction

Intensive fish farming favors the development of infectious diseases and consequently the use of antimicrobial agents for the treatment of bacterial infections has increased. One of the consequences of this practice is the selection of resistant bacteria carrying a wide variety of antimicrobial resistance encoding genes (Miranda, 2012). Although Chile is the second largest salmon producer in the world, only a few studies concerning this problem have been developed, mostly studying the occurrence of genetic determinants encoding for resistance to oxytetracycline and florfenicol (Miranda et al., 2003; Fernández-Alarcón et al., 2010; Buschmann et al., 2012), the most used drugs in Chilean salmon farms, accounting for the 99.3% of the antimicrobials used in this industry during 2016 (SERNAPESCA, 2017).

The salmon farming industry in Chile used a high amount of antibiotics, and among these, potentiated sulfonamides were included. Evidence exists that these antimicrobial agents are accumulated in the environments during treatments, leading to a change in the native bacterial composition. Thus, the selection of antibiotic resistant bacteria carrying different resistance genes is expected. In the case of those inserted in plasmids and/or integrons, they can be disseminated to susceptible bacteria in different environments (Cabello et al., 2016; Watts et al., 2017).

Despite the fact that sulfonamides are not longer authorized for used in Chilean salmon farms they were extensively used prior to introduction of drug use regulations. According to the SERNAPESCA reports (SERNAPESCA, 2011), 2009 was the last year when potentiated sulfonamides were used in the Chilean salmon industry, mainly to treat the pathogens *Flavobacterium psychrophilum* and *Streptococcus phocae*, but using only very low quantities, corresponding to the 0.01% (2008 and 2009) and 0.02% (2007) of the total amount of drug used (SERNAPESCA, 2011; Miranda et al., 2018). Furthermore, despite sulfonamides were banned for their use in Chilean salmon industry many years ago, the presence of bacteria carrying genes encoding for their resistance could be still prevalent in Chilean salmon farm environments considering the feasibility of their co-selection when other antibacterial agents such as where florfenicol or oxytetracycline are administered in fish farms.

The most frequent mechanism of bacterial resistance to sulfonamides is the acquisition of the dihydropteroate synthase enzyme encoded by the genes *sul1, sul2* and *sul3* (Grape et al., 2003; Perreten and Boerlin, 2003; Bean et al., 2009; Vinué et al., 2010). While the *sul1* gene is mainly associated with class 1 integrons, the *sul2* gene has been detected on various plasmids but not associated with integrons (Bean et al., 2009; Vinué et al., 2010), whereas the *sul3* gene has been linked to class 1 integrons lacking their 3′ CS region (Bean et al., 2009). Integrons are composed of two very conserved DNA regions, located at their ends, which are known as 5′ CS and 3′ CS (5′ and 3′ conserved segments), and between both zones one or more antibiotic resistance genes could be inserted (Ploy et al., 2000; Cambray et al., 2010; Deng et al., 2015). Acquisition and dissemination of these genes located within the integron structure results in an increase in antimicrobial resistance. Three classes of integron structure have been described (Cambray et al., 2010; Deng et al., 2015). Class 1 integrons are of principal importance in clinical isolates. The 5′-CS of class 1 integrons includes an *intI1* gene, which encodes for a specific recombinase. This gene contains the *att1* recombination site, required for specifically integrating gene cassettes. The 3′-CS region contains several open reading frames (ORFs). These include *qacEΔ1*, which confers resistance to quaternary ammonium compounds, often associated with antiseptics, along with a *sul1* gene expressing resistance to sulfonamides (Paulsen et al., 1993; Partridge et al., 2009). Resistance to trimethoprim is mainly mediated by a dihydrofolate reductase enzyme encoded by the *dfr* genes, which are usually associated with class 1 and class 2 integrons or plasmids (Yu et al., 2004; Ho et al., 2009).

This association of *sul* and *dfr* genes with mobile genetic elements such as plasmids and integrons, is highly relevant for the increase in the emergence, evolution, and dissemination of sulfonamide resistance in aquatic environments. It has been extensively reported that a high number of strains resistant to florfenicol and oxytetracycline usually exhibit simultaneous resistance to sulfonamides (Miranda and Zemelman, 2002; Miranda and Rojas, 2007; Fernández-Alarcón et al., 2010), currently the most used drugs in the Chilean salmonid industry (SERNAPESCA, 2017).

In a recent study some sulfonamide-resistance genes were studied among several isolates recovered close to a Chilean salmon farm. A low number of isolates carrying a *sul1* gene and positive for the *intI1* integrase gene were detected, but their ability to be transferred was not detected (Shah et al., 2014). Considering the role of environmental bacteria as reservoirs of antimicrobial resistance genes and the importance of salmon farming in Chile, in this study, we aimed to investigate the occurrence of *sul* and *dfr* genes, their relationship with integrons, as well as assess their capacity to be transferred by conjugation among sulfonamide- and/or trimethoprim-resistant strains recovered from various sources of Chilean salmon farms.

## Materials and Methods

### Bacterials Strains

**FIGURE 1:**
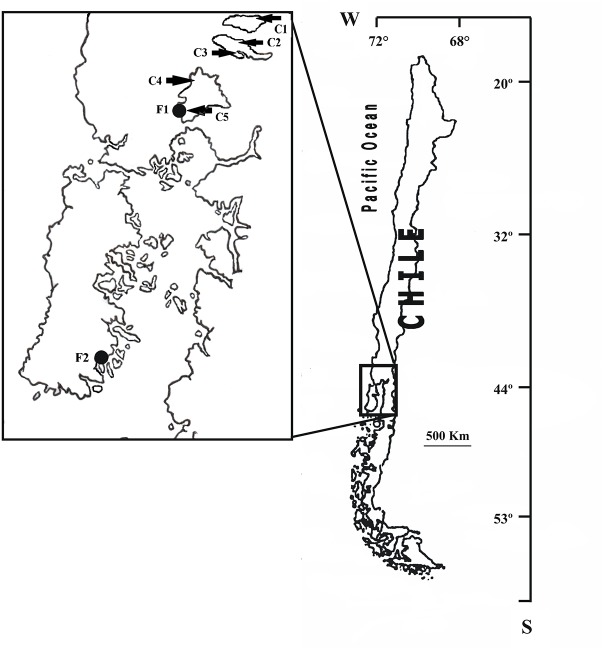
Geographic location of Chilean salmon farms, where sulfonamide- and/or trimethoprim-resistant strains were recovered, including land-based farms (F1–F2) and lake-based farms (C1–C5).

The study included 91 Gram-negative bacilli strains resistant to sulfisoxazole and/or trimethoprim recovered from a collection of bacteria obtained from seven salmon farms located in the South of Chile where the Chilean salmonid industry is concentrated (Figure 1). Fourteen strains were recovered from the land-based farms (facilities for salmonid growth until smoltification carried out in tanks located in land-based hatcheries), F1 and F2 (7 strains from each farm), and 77 strains were recovered from the lake-based farms (salmonids raised in fish cages located in freshwater lakes), C1 (6 strains), C2 (7 strains), C3 (32 strains), C4 (14 strains), and C5 (18 strains). Land-based salmonid farms F1 and F2 were located near Lake Llanquihue and Quellón Bay, respectively, whereas lake-based farms were located in the Lake Puyehue (C1), Lake Rupanco (C2 and C3) and Lake Llanquihue (C4 and C5) (Figure 1).

Strains from land-based culture centers were isolated from various sources, including un-medicated fish food pellet, salmonid fingerling and water samples from fish farm effluent and fish rearing tanks. Strains recovered from lake-based salmonid cultures were isolated from samples of mucus and intestinal content of reared fish, surface water samples from salmon cages and un-impacted control sites, as well as samples of sediments beneath salmonid cages and control sites. Samples were collected and processed as previously described (Miranda and Zemelman, 2002; Miranda and Rojas, 2007). Purified strains were stored at −85°C in CryoBank (Mast Diagnostic) vials until use.

### Bacterial Identification

Phenotypical characteristics, Gram stain, oxidase production and oxidative/fermentative utilization of glucose were determined for the 91 studied strains. The Gram reaction of strains was determined using the Gram stain (Barrow and Feltham, 1993), oxidase production was determined by the method described by Barrow and Feltham (1993), and the oxidative/fermentative (O/F) test was carried out according to Hugh and Leifson (1953). Strains were further characterized by using the GN Microplate system (Biolog, Inc., Hayward, CA, United States) according to Miranda and Rojas (2007) and a number of strains (20%) were reexamined to check reproducibility of the assay. Bacterial identification was performed by using the Microlog System 4.2 identification software (Biolog, Inc.).

The identity of thirty sulfisoxazole- and/or trimethoprim-resistant strains positive for any of the studied genes was confirmed by bacterial 16S rRNA gene sequence analysis. For DNA extraction, the bacterial strains were cultured in Tryptic Soy Agar (TSA, Difco labs) for 2 days, and total genomic DNA was extracted from each strain using the PureLink genomic DNA kit (Invitrogen). The extracted DNA was amplified by polymerase chain reaction (PCR) using the eubacterial 16S rDNA primers 27F (5′-AGAGTTTGATCCTGGCTCAG-3′) and 1492R (5′-GGTTACCTTGTTACGACTT-3′) described by Lane (1991) and PCR products were purified using the Wizard SV Gel kit and PCR Clean-up System (Promega). Sequencing of amplicons was performed by Macrogen using the ABI PRISM 373 DNA Sequencer (Applied Biosystems) with ABI PRISM cycle sequencing kit (Macrogen, South Korea). Primers 27F (5′-AGAGTTTGATCMTGGCTCAG-3′), 907R (5′-CCGTCAATTCCTTTRAGTTT-3′) and 1492R (5′-TACGGYTACCTTGTTACGACTT-3′) were used for sequencing. Sequence data was aligned and compared with available standard sequences of bacterial lineage in the GenBank database (NCBI) using Basic Local Alignment Search Tool. The partial sequences of the 16S rDNA gene belonging to each strain were deposited in the GenBank database under accession numbers KX279647 to KX279675, and MH424518.

### Minimum Inhibitory Concentrations (MICs)

Minimum inhibitory concentrations (MICs) of sulfisoxazole and trimethoprim against all strains were determined by an agar dilution method, as recommended by the Clinical and Laboratory Standards Institute (CLSI) guideline M07-A9 (CLSI, 2012a). A serial twofold dilution pattern of the antibiotic was added into Mueller–Hinton agar (Difco) so as to obtain final concentrations ranging from 0.5 to 2,048 μg mL^−1^. Bacterial suspensions were prepared in sterile 0.85% saline and triplicate plates were inoculated using a Steers replicator apparatus (Steers et al., 1959), delivering approximately 10^4^ colony-forming units per spot, and incubated for 48 h at 22°C. The first and last agar plates did not contain any antibiotic in order to detect possible contamination of the strains or antibiotic carryover. MIC was defined as the lowest concentration of the antibacterial agent producing absence of growth at least in two of the three plates after 48 h. Reference strain *Escherichia coli* ATCC 25922, recommended by CLSI (CLSI, 2012a), was used as quality control organism for verification of MIC ranges on Mueller–Hinton agar plates.

### Antimicrobial Resistance Patterns

Sulfisoxazole- and/or trimethoprim-resistant strains were tested for their susceptibility to 15 antimicrobials by an agar disk diffusion method as described in the CLSI guideline VET03-A (CLSI, 2006), using Müeller–Hinton agar (Difco). The antibacterial susceptibility patterns of resistant strains were performed using disks containing the antibacterial agents: amoxicillin (AML, 25 μg), cefotaxime (CTX, 30 μg), streptomycin (S, 10 μg), kanamycin (K, 30 μg), gentamicin (CN, 10 μg), erythromycin (E, 15 μg), chloramphenicol (CM, 30 μg), florfenicol (FFC, 30 μg), oxytetracycline (OT, 30 μg), oxolinic acid (OA, 2 μg), flumequine (UB, 30 μg), enrofloxacin (ENR, 5 μg), furazolidone (FR, 100 μg), sulfisoxazole (SFX, 300 μg), and trimethoprim (TMP, 5 μg). All disks were obtained from Oxoid, Ltd. (Basingstoke, Hampshire, England). Bacterial strains were suspended in sterile 0.85% saline to a turbidity to match a McFarland No. 2 standard (bioMerieux S.A.), diluted 1:20, and streaked on the used media. Plates were incubated for 24–48 h at 22°C and *E. coli* ATCC 25922 was used as the control strain, as recommended by the CLSI (CLSI, 2012b). Characterization of strains as resistant was stated according to standards suggested by CLSI (CLSI, 2008) or by Miranda and Rojas (2007). A number of strains (20%) were re-examined to check reproducibility of the assay.

### Detection of *sul* and *dfr* Genes

The detection of genes encoding for resistance to sulfonamide (*sul1, sul2*, and *sul3*) and trimethoprim (*dfrA1, dfrA5, dfrA6, dfrA7, dfrA8, dfrA9, dfrA10, dfrA12, dfrA13, dfrA14, dfrA17, dfrA20, dfrB1, dfrB2, dfrB3*) was made using PCR using specific primers previously described (Grape et al., 2003; Perreten and Boerlin, 2003) or designed in this study (Tables 1, 2). Colonies from an overnight culture on Mueller Hinton agar at 22°C were resuspended in 200 μL of 5% Chelex (BioRad) and 25 μL of 20 mg mL^−1^ proteinase K (Invitrogen) were added. The suspension was incubated for 45 min at 56°C followed by 8 min at 100°C, then centrifuged at 14,000 rpm and the supernatant was used as template. The PCR mixtures (25 μL of total volume) consisted of 2.5 μL of Taq reaction buffer 10× (Invitrogen), 0.75 μL of MgCl_2_ (50 mM, Invitrogen), 0.25 μL dNTPs (25 mM each, Invitrogen), 0.125 μL of each primer (25 pmoL μL^−1^, Invitrogen), 0.2 μL Taq polymerase (5 U μL^−1^, Invitrogen), and 2 μL of template DNA.

**Table 1:** Primers used to detect *sul* genes.

Gene	Primer	Sequence (5′–3′)	Amplicon size (bp)	References
*sul1*	Sul1F Sul1R	GTATTGCGCCGCTCTTAGAC CCGACTTCAGCTTTTGAAGG	408	Rosser and Young, 1999
*sul2*	Sul2F Sul2R	GAATAAATCGETCATCATTTTCGC CGAATTCTTGCGGTTTCTTTCAGC	810	Grape et al., 2003
*sul3*	Sul3F Sul3R	GAGCAAGATTTTTGGAATCG CATCTGCAGCTAACCTAGGGCTTTGG	790	Perreten and Boerlin, 2003

**Table 2:** Primers used to detect *dfr* genes.

	Primer	Sequence (5′–3′)	Amplicon size (bp)	References
*dfrA1*	D1	ACGGATCCTGGCTGTTGGTTGGAC	257	Lee et al., 2001
	D2	GCAATTCACCTTCCGGCTCGATGTC		
*dfrA5^∗^*	D3	GTTGCGGTCCAGACATAC	253	Lee et al., 2001
*dfrA14^∗^*	D4	CCGCCACCAGACACTA		
*dfrA6*	DfrA6-F	ATATCTCTTATGGCAGCTGTTTCC	420	This study
	DfrA6-R	ACTTTGCTCAAATGTTTTGCTTG		
*dfrA7*	DfrA7-F	TCTGCAACGTCAGAAAATGG	404	This study
	DfrA7-R	TGCTCAAAAACCAAATTGAAA		
*dfrA8*	D5	TCGAGCTTCATGCCATTT	454	Lee et al., 2001
	D6	TCTTCCATGCCATTCTGC		
*dfrA9*	D11	CAGATTCCGTGGCATGAACC	390	Toleman et al., 2007
	D12	GACCTCAGATACGAGTTTCC		
*dfrA10*	DfrA10-F	TATCACTTATCTTTGCGA	537	Grape et al., 2005
	DfrA10-R	GGCACCCCAACCAGCGAA		
*dfrA12*	DfrA12-F	TTTATCTCGTTGCTGCGATG	457	This study
	DfrA12-R	TAAACGGAGTGGGTGTACGG		
*dfrA13*	DfrA13-F	TCGGAAAAGAGGGAAAGATG	401	This study
	DfrA13-R	GGAGGTCTCCCCTCCTACCT		
*dfrA17*	DfrA17-F	CCGCTTAATCGGTAGTGGTC	432	This study
	DfrA17-R	TTTTTCCAAATCCGGAATGTAT		
*dfrA20*	D15	GGGAAACACCGAGAAATGGG	407	Toleman et al., 2007
	D16	TTCTTCTTCCCATTCTCCCC		
*dfrBl, dfrB2*	Dfr11-F	GATCACGTGCGCAAGAAATC	141	Navia et al., 2003
*dfrB3^∗∗^*	Dfr11-R	AAGCGCAGCCACAGGATAAAT		

*^∗^Primers used to amplify dfrA5 gene also amplify the dfrA14 gene. ^∗∗^Primers used to amplify dfrB1 gene also amplify the dfrB2 and dfrB3 genes.*

Polymerase chain reaction products were detected by electrophoresis in 1.5% agarose gels and visualized by ultraviolet illumination after staining with ethidium bromide (0.5 mg L^−1^).

### Detection and Characterization of Class 1 and Class 2 Integrons

The occurrence of class 1 and class 2 integrons was detected using PCR, amplifying intragenic fragments using specific primers for each gene (Table 3), according to previously described methodologies (Rosser and Young, 1999; Mazel et al., 2000). PCR products for *intI1* (class 1 integron) gene were confirmed using restriction enzymes, considering that SphI enzyme produces two fragments (393 and 499 bp), whereas no strains positive for *intI2* were found. Additionally, in strains carrying class 1 integrons their whole integron gene cassettes were amplified using a combination of pair of primers which align in the zone of the integrase gene and the possible gene inserted as cassette in reverse. Similarly, combinations of primers which amplify the possible gene inserted as cassette and primers which align in the 3′ CS end of the integron were used. The obtained amplification products were purified by using Wizard PCR Preps (Promega) and sequenced using the Macrogen, United States sequencing service.

**Table 3:** Primers used for PCR detection of integrons and associated gene cassette regions.

Integron	Primer	Sequence (5′–3′)	References
Class 1	IntA	GTCAAGGTTCTGGACCAGTTGC	Rosser and Young, 1999
	IntB	ATCATCGTCGTAGAGACGTCGG	
	CASS1	TGATCCGCATGCCCGTTCCATACAG	Rosser and Young, 1999
	CASS2	GGCAAGCTTAGTAAAGCCCTCGCTAG	
	5′ CS	GGCATCCAAGCAGCAAG	Lévesque et al., 1995
	3′ CS	AAGCAGACTTGACCTGA	
	qacF	GGCTGGCTTTTTCTTGTTATC	Mazel et al., 2000
	qacR	TGAGCCCCATACCTACAAAGC	
	Orf4	CTAGCGAGGGCTTTACTAAGCTTGCC	Rosser and Young, 1999
Class 2	Intl2F	GCAAATGAAGTGCAACGC	Mazel et al., 2000
	Intl2R	ACACGCTTGCTAACGATG	

### Isolation of Plasmid DNA

Bacterial strains (30 strains) harboring a studied gene (*sul1*, *sul2*, *dfrA1*, *dfrA12*, *dfrA14*, and *intI1*) were screened for their plasmid content. The plasmid isolation was made using a geneJET Plasmid miniprep kit (Thermo Scientific) according to the manufacturer’s instructions. The plasmid DNA obtained was run on 1.5% agarose gel electrophoresis for plasmids less than 20 kb and 0.8% agarose gel for plasmid greater than 20 kb. Gels were stained with GelRed^TM^ (Biotium) and viewed by UV transillumination. The size was estimated by comparing with known plasmid weight standards and standard molecular weight markers.

### Mating Experiments

Twenty-two strains were selected according their resistance pattern, MIC levels and carriage of *sul* and/or *dfr* genes and integrons, to evaluate their ability to transfer these determinants by conjugation in liquid medium according to the methodology described by Adams et al. (1998). A mutant of *E. coli* K-12 resistant to nalidixic acid, rifampicin and sodium azide was used as the recipient. Transconjugants were selected on MacConkey agar plates containing sodium azide (300 μg mL^−1^) and sulfisoxazole (512 μg mL^−1^) and tested for the presence of the antimicrobial resistance genes by PCR as previously described.

## Results

### Bacterial Identification

Out of the 91 Gram-negative bacilli strains studied, 85 (93.4%) were glucose non-fermenting bacteria, and only 6 (18.6%) were glucose fermenters. Most of the strains belonged to the *Pseudomonas* genus (81 strains), with a high frequency of *P. fluorescens* and *P. putida* (21 and 18 strains, respectively), whereas strains belonging to other non-fermenting genre included *Acinetobacter johnsonii*, *Brevundimonas vesicularis* and *Sphingobacterium multivorum* and were isolated at low frequency (Table 4). The glucose-fermenting strains were all recovered from lake-based farms and were identified as *Citrobacter gillenii*, *C. freundii*, *Kluyvera intermedia*, *Comamonas* sp., *Hafnia* sp., and *Raoultella terrigena* (Table 4). When the 30 sulfonamide- and/or trimethoprim-resistant strains positive for any of the assayed genes were identified by amplifying their 16S rRNA genes, a high predominance of representatives of the *Pseudomonas* genus (22 strains), mainly belonging to the *P. fluorescens* (6), *P. putida* (6), and *P. baetica* (3) species was observed, whereas most of the enteric strains included in the study, identified as *C. gillenii*, *C. freundii*, *Hafnia* sp., *Comamonas* sp. and *K. intermedia*, harbored at least one of the detected genes (Table 4).

**Table 4:** Identification of resistant strains recovered from Chilean salmonid farms.

Species	Number of strains							Total
	Farm							
	Land-based		Lake-based					
	F1	F2	C1	C2	C3	C4	C5	
*Acinetobacter johnsonii*		2						2
*Brevundimonas vesicularis*			1					1
*Citrobacter freundii*						1		1
*Citrobacter gillenii*			1				1	1
*Comamonas* sp.							1	1
*Hafnia* sp.							1	1
*Kluyvera intermedia*			1					1
*Pseudomonas aeruginosa*		1						1
*Pseudomonas arsenicoxydans*								1
*Pseudomonas baetica*				1	1	1		3
*Pseudomonas fluorescens*	4	2	3	2	2	4	4	21
*Pseudomonas jessenii*					1	1		2
*Pseudomonas gessardii*	1							1
*Pseudomonas lini*						1		1
*Pseudomonas ludensis*					3			3
*Pseudomonas lurida*					1			1
*Pseudomonas maculicola*					4			4
*Pseudomonas migulae*		1						1
*Pseudomonas nitroreducens*					1		1	2
*Pseudomonas oryzihabitans*						1		1
*Pseudomonas putida*	1				8	2	7	18
*Pseudomonas* sp.	1			2	5			8
*Pseudomonas synxantha*					2	1		3
*Pseudomonas syringae*		1					1	2
*Pseudomonas veronii*				1				1
*Pseudomonas viridilivida*					4		2	6
*Pseudomonas vranovensis*						1		1
*Raoultella terrígena*				1				1
*Sphingobacterium multivorum*							1	1
**Total**	**7**	**7**	**6**	**7**	**32**	**14**	**18**	**91**

### Minimum Inhibitory Concentrations (MICs)

Resistant strains exhibited high MIC values of sulfisoxazole and trimethoprim, not observing remarkable differences among strains from different farms and sources (Table 5). Sulfisoxazole MIC values of strains varied between 64 and ≥4,016 μg mL^−1^, with MIC_50_ and MIC_90_ values of 2,046 and ≥ 4,096 μg mL^−1^, respectively, whereas trimethoprim MIC values ranged from 4 to ≥2,048 μg mL^−1^, with MIC_50_ and MIC_90_ values of 2,046 and ≥2,046 μg mL^−1^, respectively (Table 5). Thus, strains showed high levels of resistance, considering that breakpoint values for categorizing resistance for these antibacterials are 16 μg mL^−1^ for trimethoprim and 512 μg mL^−1^ for sulfisoxazole (CLSI, 2015). Reference strain *E. coli* ATCC 25922, used for quality control exhibited MIC values of sulfisoxazole and trimethoprim of 8 and 0.5 μg mL^−1^, respectively, which agrees with the values recommended by CLSI (2015).

**Table 5:** Sulfixosazole (SFX) and trimethoprim (TMP) minimum inhibitory concentrations (MIC, in μg mL^−1^) of resistant strains recovered from Chilean salmonid farms.

Farm	Number of Strains	SFX			TMP		

		MIC_50_	MIC_90_	Range	MIC_50_	MIC_90_	Range
Land-based							
F1	7	512	>4,096	64 −> 4,096	>2,048	>2,048	256 −> 2,048
F2	7	>4,096	>4,096	4,096 −> 4,096	>2,048	>2,048	2,048 −> 2,048
Lake-based							
C1	6	>4,096	>4,096	>4,096	2,048	>2,048	2,048 −> 2,048
C2	7	4,096	>4,096	512 −> 4,096	2,048	> 2,048	64 −> 2,048
C3	32	512	2,048	64 −> 4,096	2,048	>2,048	256 −> 2,048
C4	14	>4,096	>4,096	512 −> 4,096	2,048	>2,048	512 −> 2,048
C5	18	4,096	>4,096	128 −> 4,096	2,048	>2,048	4 −> 2,048
**Total**	**91**	**2,048**	>**4,096**	**64 −> 4,096**	**2,048**	>**2,048**	**4 −> 2,048**

### Antimicrobial Resistance Patterns

**FIGURE 2:**
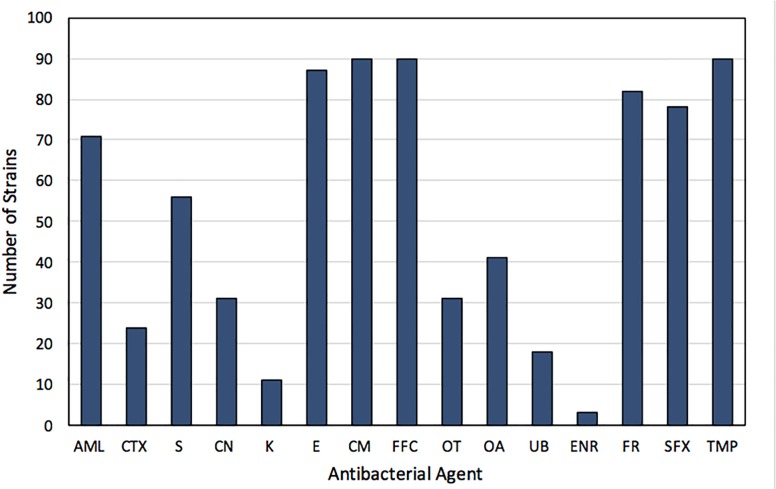
Frequency of resistance to antibacterials of 91 sulfonamide- and/or trimethoprim-resistant strains isolated from Chilean salmon farms. AML, amoxicillin; CTX, cefotaxime; S, streptomycin; CN, gentamicin; K, kanamycin; E, erythromycin; CM, chloramphenicol; FFC, florfenicol; OT, oxytetracycline; OA, oxolinic acid; UB, flumequine; ENR, enrofloxacin; FR, furazolidone; SFX, sulfisoxazole; TMP, trimethoprim.

Strains used in the study were resistant to sulfisoxazole (85.7%) and/or trimethoprim (98.9%). A high percentage of strains exhibited resistance to chloramphenicol and florfenicol (98.9%), erythromycin (95.6%) and furazolidone (90.1%). In addition, a high incidence of resistance to amoxicillin (78.0%), as well as an intermediate resistance to oxolinic acid (45.1%), oxytetracycline (34.1%), gentamicin (34.1%), and cefotaxime (26.4%) was observed in the strains (Figure 2). Otherwise, a low incidence of resistance to enrofloxacin (3.3%), kanamycin (12.1%), and flumequine (19.8%), was detected among the studied strains (Figure 2). None of the strains was susceptible to less than four antimicrobial agents. Moreover, 51.9% and 26.7% of the strains showed simultaneous resistance to 7 and 8 antibacterial agents, respectively. Among the 13 strains exhibiting susceptibility to sulfisoxazole, were those recovered from farms F1 (2), C3 (9), and C5 (2), whereas the strain susceptible to trimethoprim was recovered from farm C5.

### Detection of *sul* and *dfr* Genes

**FIGURE 3:**
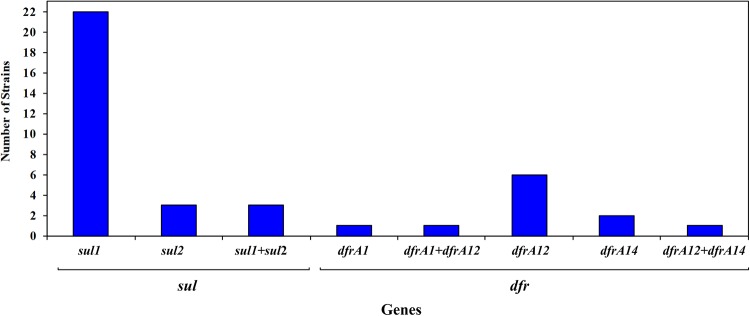
Frequency of *sul* and *dfr* genes among 91 sulfonamide- and/or trimethoprim-resistant strains isolated from Chilean salmon farms.

An important number of the sulfonamide-resistant strains (28 out of 78 strains; 35.9%) carried a *sul* gene, and among them 18 strains carried a class 1 integron (Figure 3). Among these, 22 harbored the *sul1* gene, 3 carried the *sul2* gene and 3 strains carried both genes (Figure 3). The *sul1* gene was detected in strains recovered from all 7 salmon farms, distributed in 8 out of 12 (66.7%) and 17 out of 66 (25.8%) from land-based and lake-based farms, respectively, whereas *sul2* was found only in strains from four farms (Figure 3). On the other hand, none of the studied strains carried the *sul3* gene. The majority of *sul*-positive strains were found among the strains exhibiting the highest MICs of sulfisoxazole; considering that 20/22 strains carrying *sul1* as well as 4 strains harboring *sul2* had MIC values of ≥4,098 μg mL^−1^ (Supplementary Table S1). It must be noted that 18 strains recovered from surface water (3 strains) and sediments (15 strains) from non-aquaculture unpolluted sites but exhibiting resistance to sulfisoxazole and/or trimethoprim were included in the study, but none of them were positive for any of the assayed genes.

When the presence of *dfrA* and *dfrB* genes was investigated, only the *dfrA1*, *dfrA12*, and *dfrA14* genes were detected, distributed in 12 of the 90 trimethoprim-resistant strains, with a predominance of *dfrA12* gene (8/12 strains). The distribution of *dfr* genes was similar in strains isolated from land-based (13.6%) and lake-based (11.7%) farms. In addition, it must be noted that two strains belonging to the *Citrobacter* genus, simultaneously carried two different genes encoding for resistance to trimethoprim (*C. gillenii* FP75 carried *dfrA1* and *dfrA12* genes and strain *C. freundii* FB98 carried *dfrA12* and *dfrA14* genes) (Figure 3).

### Detection and Characterization of Integrons

In this study, 21 out of 91 strains (23.1%) harbored a class 1 integron, whereas *intl2* integrase was not detected among the studied strains. This genetic structure was present in bacterial strains from land-based (6/14; 42.9%) and from lake-based (19/77; 24.7%) farms. Among strains carrying a class 1 integron, 17 strains showed a 3′ CS end with the *qacEΔ1* gene adjacent to the *sul1* gene, but 4 of them had a modified gene *qacEΔ1*, whereas in another four strains positive for the integrase *intI1* it was not possible to amplify the 3′ CS end of their integrons. As observed with strains carrying the *sul* and *dfr* genes, the integrons were concentrated in strains with high levels of resistance to trimethoprim and/or sulfisoxazole (Table 6). An important number of strains (17 strains) carried a *sul* gene inserted in integrons (Table 6).

**Table 6:** Identification, integron structure, location of *sul* and *dfr* genes and plasmid content among sulfonamide- and/or trimethoprim-resistant strains.

Strain	Farm	Source	Class of integron integrase	3′-CS	Gene cassettes inside the Variable Zone (From 5′ to 3′)	*sul* and *dfr* genes outside the integron	Plasmid content	
							
							Number	Size (kb)
*Pseudomonas jessenii* OT42	F1	Mucus	–	−	–	*sul1*	1	10
*Pseudomonas putida* 0233	F1	Tank water	*intI1*	+	*aadA1*	–	1	33
*Pseudomonas migulae* Q11	F2	Tank water	*intI1*	+	*aadA1*	–	0	
*Pseudomonas fluorescens* Q20	F2	Fingerling	*intI1*	+	–	–	1	25
*Pseudomonas fluorescens* Q23	F2	Fingerling	*intI1*	+	*aadA1*	–	0	
*Pseudomonas syringae* Q64	F2	Effluent	*intI1*	+	–	–	1	10
*Acinetobacer johnsonii* Q67	F2	Pellet	*intI1*	+	*aadA1*	*dfrA1, sul2*	1	10
*Acinetobacer johnsonii* Q75	F2	Pellet	*intI1*	+	*aadA1*	–	1	30
*Pseudomonas fluorescens* FP37	C1	Mucus	*intI1*	+	*aadA2, cmlA*	*dfrA12*	1	10
*Pseudomonas fluorescens* FP45	C1	Mucus	*intI1*	+	*aadA2, cmlA*	*dfrA12*	1	10
*Pseudomonas fluorescens* FP47	C1	Mucus	*intI1*	+	*aadA2, cmlA*	*dfrA12*	1	30
*Citrobacter gillenii FP75*	C1	Mucus	*intI1*	+	*dfrA12, orfF, aadA2a*	*dfrAl, sul2*	1	10
*Kluyvera intermedia* OP29	C1	Cage sediment	*intI1*	+	*dfrA12, orfF, aadA2*	*sul2*	1	10
*Pseudomonas baetica* FE3	C2	Cage water	–	−	–	*sul1*	2	0.25/25
*Pseudomonas veronii* FE4	C2	Cage water	–	−	–	*sul1*	0	
*Pseudomonas putida* FF32	C3	Cage water	*intI1*	−	–	–	1	10
*Pseudomonas baetica* SX52	C3	Mucus	*intI1*	+	*aadA9*	*dfrA12*	1	30
*Pseudomonas putida* FB13	C4	Mucus	–	−	–	*sul1*	1	30
*Pseudomonas putida* FB 15	C4	Mucus	*intI1*	−	–	*dfrA14, sul1*	1	10
*Citrobacter freundii* FB98	C4	Mucus	*intI1*	+	*dfrA12, orfF, aadA2*	*dfrA14*	1	3
*Pseudomonas fluorescens* FR27	C4	Mucus	*intI1*	+	*aadA2, cmlA*	*dfrA12*	2	10/40
*Pseudomonas baetica* FR34	C4	Mucus	–	−	–	*sul2*	2	0.6/25
*Pseudomonas jessenii* FR51	C4	Mucus	–	−	–	*sul1*	0	
*Pseudomonas arsenicoxydans* SR72	C4	Intestinal content	–	−	–	*sul1*	1	25
*Sphingobacterium multivorum* FM2	C5	Cage water	–	−	–	*sul2*	1	40
*Comamonas* sp. FM3	C5	Cage water	*intI1*	+	*aadA2*	–	2	1/10
*Pseudomonas syringae* FM4	C5	Cage water	*intI1*	+	–	–	2	10/37
*Hafina sp*. FM7	C5	Cage water	*intI1*	−	–	–	2	2.5/10
*Pseudomonas putida* FM15	C5	Mucus	*intI1*	−	–	*sul2*	1	30
*Pseudomonas putida* FM22	C5	Mucus	–	−	–	*sul1, dfrA14*	1	10

*3′ CS, 3′ conserved segment of class-1 integrons harboring the gene array qacEΔ1 + sul1.*

Fourteen variable zones were found among the detected class 1 integrons, carrying 1 to 3 gene cassettes. Five genes encoding for resistance to trimethoprim (*dfrA12*), aminoglycosides (*aadA1*, *aadA2*, and *aadA9*), and chloramphenicol (*cmlA*) were identified as part of the integron structures, being found either alone or in combination with other gene cassettes (Table 6). The variable regions with one cassette harbored the genes *aadA1* (five strains), *aadA2* (one isolate) and *aadA9* (one isolate). The strains with two cassettes inserted in its variable region carried *aadA2*-*cmlA* (four strains). Finally, three strains were found to carry three gene cassettes in their variable zones containing the genes *dfrA12-orf-aadA2* (two strains) and *dfrA12-orf-aadA2a* (one strain). Otherwise, in three strains the variable zone of their integrons could not be determined, despite using various primer combinations (Table 6).

**Table 7:** Transfer of *sul* and *dfr* genes and integrons from resistant strains to *Escherichia coli* K-12.

Donor	Transconjugant				Frequency of transfer^∗^
	Integron	Cassette	Genes out of VZ	Resistance pattern	
FB98	1	*dfrA12, orfF, aadA2*	–	S-CM-FFC-OT-NA-SFX-TMP-NaN_3_	7.4 × 10^−3^
FM4	1	–	–	S-CM-FFC-OT-NA-SFX-NaN_3_	1.4 × 10^−3^
FM7	1	–	–	NA-TMP-NaN_3_	5.4 × 10^−3^
FM22	–	–	*sul1, dfrA14*	S-CM-FFC-OT-NA-SFX-TMP-NaN_3_	2.6 × 10^−5^
FP45	1	*aadA2, cmlA*	–	S-CM-NA-TMP-NaN_3_	4.1 × 10^−3^
FP75	1	*dfrA12, orfF, aadA2*	*sul2*	S-CM-FFC-OT-NA-SFX-TMP-NaN_3_	9.8 × 10^−4^
OP29	1	*dfrA12, orfF, aadA2*	*sul2*	S-CM-FFC-OT-NA-SFX-TMP-NaN_3_	8.4 × 10^−3^
SX52	1	*aadA9*	*dfrA12*	S-NA-TMP-NaN_3_	5.0 × 10^−4^
OT42	–	–	*sul1*	S-NA-TMP-NaN3	1.7 × 10^−3^
FR27	1	*aadA2, cmlA*	*dfrA12*	S-CM-FFC-NA-TMP-NaN_3_	3.1 × 10^−5^
FE3	NT				
FM2	NT				
FM3	NT				
Q20	NT				
Q64	NT				
Q67	NT				
Q75	NT				
0233	NT				
FB13	NT				
FB15	NT				
FR34	NT				
SR72	NT				

*VZ, variable zone; ^∗^transconjugant per recipient cell; S, streptomycin; CM, chloramphenicol; FFC, florfenicol, OT, oxytetracycline; NA, nalidixic acid; SFX, sulfisoxazole; TMP, trimethoprim; NaN_3_, sodium azide; NT, not transfer detected.*

It must be noted that various strains harboring a class 1 integron carried genes encoding for resistance to sulfonamides and trimethoprim not inserted in the integron structure. For the *dfr* genes two situations were seen, the presence of *dfr* genes in strains with integrons, but not associated with these structures, and the presence of more than one *dfr* gene per strain (associated and not associated with integron). In the case of *sul* genes, these were found mainly associated with class 1 integrons, with *sul1* as part of the integron, whereas *sul2* was not present as a gene cassette, but rather associated with other structures (Table 6).

The *dfrA12* gene encoding for the enzyme dehidrofolate reductase XII, carried by the FP75, FB98 and OP29 strains, was analyzed using the 5′ CS/dfrA12 R, dfrA12 F/3′ CS and dfrA12F/sul1R primers, and the cassette was found to be located adjacent to the integrase gene. In addition, when amplicons were sequenced, two cassettes were detected, corresponding to the *orf* and *aadA2a* genes (FP75 strain), and *orf* and *aadA2* genes (FB98 and OP29 strains). These genes were adjacent to the *qacEΔ1* gene in the 3′ CS end, presenting a 100% similarity to the *dfrA12*, *orfF*, and *aadA2a* genes included in the GenBank database under the Accession No. DQ322593.

### Plasmid Content

Plasmid DNA was detected in 25 out of 30 resistant strains examined, and from these, six strains contained two plasmid bands. The number and size of plasmid bands found are shown in Table 6. The most commonly found plasmid band had a molecular weight of 10 kb (13 strains) followed by plasmids with a range from 25 to 40 kb (12 strains), with a predominance of plasmids of 30 kb (four strains). Among the four strains carrying two plasmids, all of them carried a 10 kb plasmid and were identified as *Hafnia* sp., *Comamonas* sp., *P. syringae*, and *P. fluorescens* (Table 6).

### Transfer of Resistance Genes

Among the 22 strains assayed for conjugation experiments, in 10 strains it was demonstrated that *sul* and *dfr* genes, as well as the integrons were transferable. A high frequency of transfer of resistance to sulfonamides was observed in the positive strains, ranging from 10^−3^ to 10^−5^ (transconjugant cells per recipient cells). The majority of these strains (6 out of 10) showed a frequency of transfer of 10^−3^, whereas only two strains showed a frequency of 10^−5^ (Table 7). In addition, strains FP75, OP29, OT42, FM7, FM22, and FB98 were able to co-transfer resistance to oxytetracycline, chloramphenicol and florfenicol, whereas strain FR27 co-transferred resistance to chloramphenicol and florfenicol. It was observed that all transconjugant strains acquired similar levels of resistance to antimicrobials to those exhibited by the donor strains.

Six strains used as donors and carrying a class 1 integron were able to transfer this structure as well as their associated gene cassettes (Table 7). Furthermore, *sul* and *dfrA* genes not associated with integrons were transferred, corresponding to the *sul1* gene carried by OT42 and FM22 strains, *sul2* gene carried by the FP75 and OP29 strains, and *dfrA12* carried by the SX52 and FR27 strains (Table 7). On the contrary, other *dfr* genes not associated with integrons such as the *dfrA12*, *dfrA14*, and *dfrA1* genes carried by the FP45, FB98 and FP75 strains, respectively were not transferred by conjugation (Table 7). The simultaneous transfer of *sul1* and *sul2* genes from strains *C. gillenii* FP75 and *K. intermedia* OP29, which carried both genes suggests the location of these genes on the same plasmid, considering that both strains carried only one plasmid. Otherwise, an important number of the assayed strains, both carrying and not carrying a class 1 integron (7 and 5 strains, respectively) were not able to transfer by conjugation their resistance to sulfonamides and/or trimethoprim, as well as the associated resistance encoding genes (Table 7).

## Discussion

In previous studies investigating resistant strains from Chilean salmon farms with different histories of antimicrobial usage the occurrence of resistance to trimethoprim/sulfametoxazole, accompanied with resistance to florfenicol and oxytetracycline, as well as the carriage of *floR* and *tet* genes respectively has been reported (Miranda and Zemelman, 2002; Miranda et al., 2003; Miranda and Rojas, 2007; Fernández-Alarcón et al., 2010). This was regardless of whether sulfonamides had been used or not, thus suggesting that sulfonamide-resistance may be selected and maintained by the use of other antimicrobials, such as florfenicol or oxytetracycline, the most used antimicrobials in Chilean salmon farms (SERNAPESCA, 2017; Miranda et al., 2018). Furthermore, Sköld (2001) concluded that susceptibility to sulfonamides and trimethoprim will not return after suspending their use, mainly due to the reported high variety of mechanisms of bacterial resistance to these antibacterials, helping to explain the finding of the persistence of these resistances even without selective pressure.

The high incidence of multiresistance exhibited by the studied strains is according with previous studies of antibiotic resistant bacteria recovered from Chilean salmon farms and sites near salmon farms (Miranda and Zemelman, 2002; Miranda and Rojas, 2007; Shah et al., 2014), in which most of strains showed simultaneous resistance to 4–10 antibacterial agents. Otherwise, MIC values of sulfisoxazole and trimethoprim of strains were remarkable high, but no previous MIC values of strains recovered from Chilean salmon farms are available. The high MIC values of these antibacterials suggest the concurrence of *sul* and *dfr* genes.

Among the *sul*-carrying strains, 19 of these strains carried the *sul1* gene associated with the 3′ conserved end, proper of a class 1 integron, as was reported by Rosser and Young (1999), whereas the occurrence of nine strains carrying the *sul1* gene not associated with an integron structure, is in accordance with that reported by Grape et al. (2005) and Infante et al. (2005) in clinical strains. On the contrary the detected *sul2* genes were not integron inserted, in agreement with Agersø and Petersen (2007), who studied *Acinetobacter* strains resistant to sulfametoxazole recovered from fish farms in Thailand. Thus, the positive transfer of *sul2* genes is consistent with a location on another mobile element, such as small size plasmids as was previously reported (Rådström and Swedberg, 1988; Enne et al., 2004; Bean et al., 2005; Agersø and Petersen, 2007). As *sul* genes were not detected in 64 sulfonamide-resistant strains, other resistance mechanisms must be involved, most probably multidrug efflux pumps, considering the multiresistant phenotypes of studied strains as well as the high predominance of non-fermenters of glucose, which usually exhibit this mechanism of resistance (Maseda et al., 2000).

In this study, the *dfrA1, dfrA12*, and *dfrA14* genes were found, with *dfrA12* the most frequent. Lee et al. (2001) reported these genes encoding for enzymes conferring the highest levels of resistance to trimethoprim, in accordance with this study, because strains carrying *dfr* genes exhibited the highest levels of resistance to trimethoprim. Otherwise, the feasibility of carrying other *dfr* genes cannot be discarded, especially in those strains negative for any of the assayed *dfr* genes and exhibiting high levels of resistance to trimethoprim. It was observed that the majority of assayed strains from F1 and F2 land-based farms (5/6) was not capable of transfer by conjugation their plasmids and *sul* or *dfr* genes, whereas an important number of strains recovered from C1 and C5 lake-based farms (6/8) was able to transfer their integrons as well as their *sul* and *dfr* genes, being included or not in a class 1 integron. Furthermore, considering that transfer of sulfonamide resistance was usually accompanied with a transfer of resistance to the antimicrobials most intensively used in Chilean salmon farms, such as florfenicol and oxytetracycline, these antibacterials might promote the selection and spread of antibiotic resistance genes encoding for sulfonamide and trimethoprim resistance via co-selection.

In a previous study, Shah et al. (2014) studied a total of 124 strains recovered from aquaculture impacted sediments located near Chilean salmon farms, founding 62 and 58 strains exhibiting resistance to sulfamethizole and trimethoprim, respectively, with 12 strains carrying the *sul1* gene and the integrase-encoding *int1* gene. Otherwise, *dfrA1, dfrA5*, and *dfrA12* genes were detected in 17, 14, and 22 strains, respectively. These authors studied a group of quinolone resistant strains recovered from site near a Chilean salmon farm, and detected three strains identified as *Dietzia* sp., *Rhodococcus* sp., and *Arcobacter* sp., to be carrying class 1 integrons with the *dfrA12*-*aadA2*-*qacEΔ1*-*sul1* array (Tomova et al., 2018), similar to those shown by the FP75, FB98 and OP29 strains, but lacking the *orfF* gene. Thus, this study reports for the first time the carriage of the gene *dfrA14*, encoding for resistance to trimethoprim as well as the genes *aadA1* and *aadA9*, encoding for resistance to aminoglycosides, among bacteria associated to Chilean salmonid farms, whereas all the observed gene arrays within class 1 integron are described for the first time among bacteria associated to Chilean salmon farming. According to the sequencing analysis, all cassette arrays found in the study have been previously described, mainly associated to enteric bacteria (Chang et al., 2007; Domingues et al., 2015), whereas the gene cassettes identified in the study were not novel.

Most of previous studies dealing with the occurrence of integrons in *Pseudomonas* considered clinical strains belonging to the *Pseudomonas aeruginosa* species. It has been reported that class 1 integrons have been found to be widespread in *P. aeruginosa* isolated from environmental and clinical settings from various countries (Fonseca et al., 2005; Gu et al., 2007; Chen et al., 2009; Ruiz-Martínez et al., 2011; Martínez et al., 2012; Kiddee et al., 2013; Kor et al., 2013; Hsiao et al., 2014; Adesoji et al., 2015; Khosravi et al., 2017), in according with the results observed in this study, in which the majority of the strains carrying a class 1 integron belonged to the *Pseudomonas* genus.

After amplifying and DNA sequencing of whole class 1 and integron gene cassettes, a total of six different types of gene cassette arrays were identified, with a predominance of *aadA1* (5 strains) and *aadA2*-*cmlA* (4 strains) (Table 6). It is interesting to note the high incidence of *aadA* genes (14 strains), encoding for the enzyme aminoglycoside adenyltransferase that confers resistance to aminoglycosides, inserted in the variable zone of a class 1 integron, considering that no aminoglycosides have been used in Chilean salmon farming, thus suggesting that these integrons have a human clinic origin. In other studies, most of the class 1 integrons identified in *E. coli* strains carried the *dfr12*-*orfF*-*aadA2* cassette array (Kang et al., 2005; Chang et al., 2007), as was observed in this study for the enteric bacteria.

Otherwise, while the occurrence of *int2* gene in *Pseudomonas aeruginosa* has been previously reported (Xu et al., 2009; Goudarzi et al., 2015), none of the *Pseudomonas* strains included in this study were found to carry the *int2* gene, which was in concordant to other reports of *P. aeruginosa* from clinical settings (Rossolini and Mantengoli, 2005; Sun et al., 2014; Khosravi et al., 2017). It must be noted that class 2 integrons are not usually detected in *Pseudomonas* strains from environmental sources, being most commonly associated with members of the family Enterobacteriaceae, such as *E. coli* and *Salmonella enterica* (Ahmed et al., 2005; Antunes et al., 2006; Cocchi et al., 2007; Essen-Zandbergen et al., 2007; Laroche et al., 2009; Ozgumus et al., 2009).

Of the class 1 integron-containing strains, it was demonstrated the occurrence of an empty integron in seven strains, and possible causes could be the lack of the 3′ conserved segment or insufficient homology to the 3′ conserved segment primer to produce a product such as the observed in strains *Hafnia* sp. FM7, *Pseudomonas putida* FM15, *P. putida* FB15 and *P. putida* FF32, but strains *P. fluorescens* Q20, *P. syringae* Q64 and *P. syringae* FM4 possessed a 3′ conserved segment (Table 6). Thus, most probably, the lack of integrated genes cassettes may be a consequence of the excision of previously integrated cassettes from the integron when antibiotic selective pressure is diluted in the environment, as was concluded by Ruiz-Martínez et al. (2011). Furthermore, these empty structures could confer to these strains the capacity to adapt rapidly to the aquaculture environment, where antibiotics are intensively used, conferring to them a selective advantage by means of the acquisition of new antibiotic resistance genes. Otherwise, it was suggested that these empty structures could be used as indicators of the absence of a sustained antimicrobial pressure (Ruiz-Martínez et al., 2011).

Otherwise, the absence of the conserved end 3′-CS in four strains carrying class 1 integrons could be a consequence of the activity of insertion sequences which could produce non-functional genes (Laroche et al., 2009; Dawes et al., 2010). Furthermore, it must be noted that integrons of environmental bacteria commonly exhibit deletions in their structures, contrary to the observed in the integrons harbored by clinical strains. In this trend, complex integrons associated with the insertion sequence IS*CR1* have been reported (Quiroga et al., 2007; Song et al., 2010), which frequently have non-cassette type resistance genes and a truncated version of 3′-CS region followed by an IS*CR1* element (Toleman et al., 2006a,b). Other studies demonstrated that rearrangements produced by the insertion sequence IS26 in the integrons may change class 1 integron structure causing the loss of parts of the 3′-CS (Labbate et al., 2008; Doublet et al., 2009; Juan et al., 2010).

The association of integrons inserted in conjugative plasmids and antimicrobial resistance was confirmed by transfer experiments for 10 out of 22 assayed strains. Therefore, integrons appear to have an essential role in facilitating the dissemination of the resistance genes and contributing to the creation of multidrug resistant phenotypes. Furthermore, previous studies demonstrated that copy numbers between the *sul1* and *intI1* genes were significantly correlated suggesting that class 1 integrons may play a role in the prevalence and propagation of *sul1* in various aquatic environments, including farm sediments and marine coastal areas (Muziasari et al., 2014; Na et al., 2014; Lin et al., 2015). It is well-known that *intI1* genes are the most abundant gene capture and transmission system in clinical and environmental strains (Partridge et al., 2009; Shah et al., 2012; Domingues et al., 2015), and their abundance and structures are greatly influenced by anthropogenic contamination (Wright et al., 2008). Considering that class 1 integrons are common among multi-drug resistant bacteria inhabiting natural environments associated with human activities, where a strong selection pressure is imposed by the use of antimicrobials, integrons have been proposed as markers for the identification of multi-drug resistant strains, as well as for anthropogenic pollution (Gillings et al., 2008; Gillings et al., 2015).

## Conclusion

The present results demonstrated that Chilean salmon farms play an important role as reservoirs of sulfonamide- and trimethoprim-resistant bacteria. The prevalence of conjugative plasmids and integrons among *sul*-carrying bacteria suggests these bacteria, mainly belonging to the *Pseudomonas* genus may contribute to high spread of bacterial resistance to sulfonamides and other antibacterials in environments associated with Chilean salmon farms. This is the first study reporting the occurrence of transferable *sul* and *dfr* genes and integrons among the antimicrobial resistant bacteria associated with Chilean salmonid farms and the results suggest that the increasing prevalence of antibiotic resistant bacteria in Chilean salmon farming resistance is partly attributable to the acquisition, dissemination and stable maintenance of a class 1 integron. Thus, a continuous surveillance of these resistance elements even in absence of antibacterial therapy is urgently required to evaluate the potential role of fish farming environments as reservoirs and source of elements composing the mobilome, such as conjugative plasmids and integrons, increasing the risk of their dissemination to human pathogens through horizontal transfer.

## Author Contributions

MD planned the experiments, analyzed the data, and participated in manuscript drafting. CM isolated and provided the studied strains, studied antimicrobial susceptibility patterns of resistant bacteria, analyzed the data, wrote the manuscript and is the corresponding author and primary contact during the manuscript submission, review, and publication process. OF performed the MIC assays, detected the genes encoding for sulfonamide and trimethoprim resistance, as well as integrase and variable region of detected integrons, and also carried out their DNA sequence analysis. MF performed conjugation assays and participated in manuscript drafting. FG identified the studied strains by sequencing their 16S rRNA genes, carried out DNA sequence analysis and determined the plasmid content of strains. HB-T and GG-R participated in manuscript drafting, revisions and interpretation of data. All authors have made intellectual contribution to the work, and approved it for publication.

## Conflict of Interest Statement

The authors declare that the research was conducted in the absence of any commercial or financial relationships that could be construed as a potential conflict of interest.
