# Supplementary material for: Occurrence of Transferable Integrons and sul and dfr Genes Among Sulfonamide-and/or Trimethoprim-Resistant Bacteria Isolated From Chilean Salmonid Farms
Source: Front Microbiol. 2019 Apr 12;10:748. doi: 10.3389/fmicb.2019.00748 (PMC6474311; doi:10.3389/fmicb.2019.00748)
Supplement: Supplementary file 1 [file Table_1.DOCX]

**TABLE S1| Sulfisoxazole (SFX) and Trimethoprim (TMP) Minimum Inhibitory Concentrations (MIC, in µg mL^-1^) and Antibiotic Resistance of Strains.**

Strain MIC SFX MIC TMP Resistance phenotype

*Pseudomonas jessenii* OT42 512 2,048 AML-CTX-CM-FFC-OT-E-FR-SFX-TMP

*Pseudomonas putida* O233 >4,096 >2,048 AML-CTX-CM-FFC-OT-E-FR-SFX-TMP

*Pseudomonas migulae* Q11 >4,096 >2,048 AML-CTX-CM-FFC-OT-E-FR-SFX-TMP

*Pseudomonas fluorescens* Q20 4,096 >2,048 AML-CTX-S-CM-FFC-OT-E-FR-SFX-TMP

*Pseudomonas fluorescens* Q23 >4,096 2,048 AML-CTX-S-CM-FFC-OT-E-FR-SFX-TMP

*Pseudomonas syringae* Q64 >4,096 >2,048 AML-CM-FFC-OT-E-FR-SFX-TMP

*Acinetobacter johnsonii* Q67 >4,096 >2,048 FFC-OT-E-FR-SFX-TMP

*Acinetobacter johnsonii* Q75 >4,096 >2,048 CM-FFC-OT-FR-SFX-TMP

*Pseudomonas fluorescens* FP37 >4,096 2,048 AML-S-K-CM-FFC-OT-E-OA-UB-FR-SFX-TMP

*Pseudomonas fluorescens* FP45 >4,096 2,048 AML-S-K-CM-FFC-OT-E-OA-UB-FR-SFX-TMP

*Pseudomonas fluorescens* FP47 >4,096 2,048 AML-CTX-S-K-CM-FFC-OT-E-FR-SFX-TMP

*Citrobacter gillenii* FP75 >4,096 >2,048 AML-S-CM-FFC-OT-E-FR-SFX-W-TMP

*Kluyvera intermedia* OP29 >4,096 >2,048 AML-S-CM-FFC-OT-E-OA-UB-SFX-TMP

*Pseudomonas baetica* FE3 512 64 AML-CTX-CM-FFC-E-FR-SFX-TMP

*Pseudomonas veronii* FE4 >4,096 2,048 AML-CTX-CM-FFC-E-OA-UB-FR-SFX-TMP

*Pseudomonas putida* FF32 64 2,048 CM-FFC-OT-E-FR-TMP

*Pseudomonas baetica* SX52 512 2,048 S-CM-FFC-OT-E-OA-FR-SFX-TMP

*Pseudomonas putida* FB13 >4,096 >2,048 AML-CTX-CM-FFC-E-OA-UB-FR-SFX-TMP

*Pseudomonas putida* FB15 >4,096 >2,048 AML-E-CM-FFC-OA-UB-FR-SFX-TMP

*Citrobacter freundii* FB98 >4,096 2,048 S-K-E-CM-FFC-OT-SFX-TMP

*Pseudomonas fluorescens* FR27 2,048 >2,048 AML-CTX-S-CM-FFC-E-OA-UB-FR-SFX-TMP

*Pseudomonas baetica* FR34 >4,096 2,048 AML-CTX-CM-FFC-E-OA-UB-ENR-FR-SFX-TMP

*Pseudomonas jessenii* FR51 >4,096 1,024 AML-CTX-CM-FFC-E-FR-SFX-TMP

*Pseudomonas arsenicoxydans* SR72 512 512 AML-CTX-CM-FFC-E-FR-SFX-TMP

*Sphingobacterium multivorum* FM2 >4,096 >2,048 S-K-CN-CM-FFC-OT-E-OA-FR-SFX-TMP

*Comamonas* sp. FM3 >4,096 32 S-CM-FFC-OT-E-SFX-TMP

*Pseudomonas syringae* FM4 >4,096 >2,048 CTX-S-CM-FFC-OT-E-OA-UB-FR-SFX-TMP

*Hafnia* sp. FM7 4,096 4 S-CM-FFC-OT-E-OA-UB-FR-SFX

*Pseudomonas putida* FM15 >4,096 >2,048 S-CN-CM-FFC-OT-E-OA-UB-ENR-FR-SFX-TMP

*Pseudomonas putida* FM22 >4,096 >2,048 CTX-S-K-CN-CM-FFC-OT-E-OA-UB-FR-SFX-TMP

*AML, amoxicillin; CTX, cefotaxime; S, streptomycin; K, kanamycin; CN, gentamicin; E, erythromycin; CM, chloramphenicol; FFC, florfenicol; OT, oxytetracycline; OA, oxolinic acid; UB, flumequine; ENR, enrofloxacin; FR, furazolidone; SFX, sulfisoxazole; TMP, trimethoprim*
